# A double-blind comparison of morphological and collagen fingerprinting (ZooMS) methods of skeletal identifications from Paleolithic contexts

**DOI:** 10.1038/s41598-023-45843-4

**Published:** 2023-11-01

**Authors:** Eugène Morin, Ellie-May Oldfield, Mile Baković, Jean-Guillaume Bordes, Jean-Christophe Castel, Isabelle Crevecoeur, Hélène Rougier, Gilliane Monnier, Gilbert Tostevin, Michael Buckley

**Affiliations:** 1 Department of Anthropology, Trent University, DNA Bldg Block C, 2140 East Bank Drive, Peterborough, ON K9J 7B8 Canada; 2 Université de Bordeaux, PACEA, Allée Geoffroy St-Hilaire CS 50023, 33615 Pessac Cedex, France; 3 School of Natural Sciences, Manchester Institute of Biotechnology, University of Manchester, Manchester, M1 7DN UK; 4 Center for Conservation and Archaeology of Montenegro, UI. Njegoseva bb., Cetinje, Montenegro; 5 Département d’Archéozoologie, Muséum d’histoire naturelle, Route de Malagnou 1, 1208 Geneva, Switzerland; 6 Department of Anthropology, California State University, Northridge, 18111 Nordhoff St., Northridge, CA 91330-8244 USA; 7 Department of Anthropology, University of Minnesota, 395 H.H. Humphrey Center, 301 19th Ave. S, Minneapolis, MN 55455 USA

**Keywords:** Anthropology, Archaeology

## Abstract

Modeling the subsistence strategies of prehistoric groups depends on the accuracy of the faunal identifications that provide the basis for these models. However, our knowledge remains limited about the reproducibility of published taxonomic identifications and how they accurately reflect the range of species deposited in the archaeological record. This study compares taxonomic identifications at three Paleolithic sites (Saint-Césaire and Le Piage in France, Crvena Stijena in Montenegro) characterized by high levels of fragmentation. Identifications at these sites were derived using two methods: morphological identification and collagen fingerprinting, the latter a peptide-based approach known as ZooMS. Using a double-blind experimental design, we show that the two methods give taxonomic profiles that are statistically indistinguishable at all three sites. However, rare species and parts difficult to identify such as ribs seem more frequently associated with errors of identification. Comparisons with the indeterminate fraction indicate that large game is over-represented in the ZooMS sample at two of the three sites. These differences possibly signal differential fragmentation of elements from large species. Collagen fingerprinting can produce critical insights on the range distribution of animal prey in the past while also contributing to improved models of taphonomic processes and subsistence behavior.

## Introduction

Most archaeological models of subsistence are informed by sampled archaeofaunal remains identified to element and taxon. Estimates of skeletal and taxonomic abundances that drive these models are often treated as accurate and representative of the range of skeletal elements and species that were initially present. However, several studies have raised problems with this assumption and noted that various metrics of skeletal and taxonomic abundances are affected by fragmentation and other processes^1–7^. Instead of revisiting the merits of different measures of faunal abundance, our goal here was to compare taxonomic identifications obtained by independent methodologies and to explore what can be learned from the observed mismatches.

Identifying faunal remains using visual and tactile cues is a complex task that requires extensive knowledge of vertebrate anatomy^8^. Identifications to taxon are generally more secure when the specimens can be compared to a reference collection that includes a wide range of species and animals from both sexes and from various age classes^9,10^. However, extensive fragmentation often impedes taxonomic identification by removing critical landmarks from the specimen and/or by reducing the cross-section that can be examined.

To circumvent problems of identification caused by fragmentation, alternatives to traditional methods based on skeletal morphology (hereafter, referred to as the morphological method) have been developed. One of these approaches is collagen fingerprinting, more accurately a form of peptide mass fingerprinting commonly referred to as ZooMS (short for Zooarchaeology by Mass Spectrometry). As the name implies, collagen fingerprinting utilizes collagen—the dominant protein in modern bone—and what is considered the longest surviving protein in ancient skeletal tissue^11^, commonly outlasting ancient DNA preservation^12^. The method relies on the successful extraction of collagen, either the acid-soluble fraction brought into solution upon the decalcification of the bone mineral^13^ and its ultrafiltration into a buffer suitable for enzymatic digest, or the acid-insoluble fraction which is then brought into solution through gelatinization directly within such a buffer^14^. The buffered collagen extract is then digested into protein fragments called peptides using an enzyme, most commonly the protease trypsin. Some portion of the resultant digest (sometimes following some form of purification) is then co-crystalised with a laser-absorbing matrix, such as alpha-cyano hydroxycinnamic acid, ready for analysis by Matrix Assisted Laser Desorption Ionization Time-Of-Flight (MALDI-ToF) mass spectrometry. The spectra that are generated represent the dominant peptides within a sample, almost entirely derived from type I collagen when the sample contains bone. These spectra are then compared with those of known reference materials, with a focus on a number of peptide biomarkers used to assess potential matches between the test sample and the reference species.

Few studies have compared the morphological and collagen fingerprinting approaches from a methodological perspective. In the morphological approach, identification should in principle proceed in a random fashion; the entire sample is tentatively identified regardless of specimen size, type or morphology (but see refs.^15^ and^9^ for other approaches to faunal identification that are focused on specific skeletal portions). As an independent method, collagen fingerprinting has considerable potential because it can help to address how the morphologically identified sample relates to the deposited assemblage. However, a relatively small proportion of remains is typically selected for collagen fingerprinting because costs and other constraints—the method is destructive (but see emerging non-destructive approaches, e.g.,^16^—limit the number of specimens that can be analyzed. One additional problem is that archaeozoologists who are selecting specimens for ZooMS may instinctively sample remains with more robust morphological identifications to avoid mismatches between the two methods. Biases toward more secure identifications are to be expected due to natural apprehensions regarding the production of errors in science^17^. Furthermore, a focus on certain classes of specimens in ZooMS sampling (e.g., specimens that are morphologically identified to taxon and rare species or species difficult to identify using the morphological approach) may produce taxonomic biases because the sampling process is no longer random in nature.

With these issues in mind, we compared morphological and collagen fingerprinting identifications from three different sites in an attempt to answer two questions: (1) do the morphological and collagen fingerprinting approaches produce comparable results?, and (2) are identifications produced by the two methods biased toward certain taxa or classes of skeletal elements? Addressing these questions may help improve our knowledge of identification biases in archaeozoological analyses and how they can impact interpretations of past subsistence activities.

## Materials and methods

Taxonomic identifications of animal remains were compared at three sites: Crvena Stijena in Montenegro and Saint-Césaire (also known as La Roche-à-Pierrot) and Le Piage in France. Located in Western France, the site of Saint-Césaire is a collapsed rockshelter containing a sequence of late Mousterian, Mousterian/Châtelperronian and Aurignacian occupations. The fauna from this site has been identified using traditional archaeozoological methods with most remains being attributed to reindeer (*Rangifer tarandus*), large bovines (*Bos*/*Bison*), horse (*Equus ferus*) and red deer (*Cervus elaphus*)^18^. Le Piage is a cliff deposit in Southwest France comprising occupations dated to the late Mousterian, Protoaurignacian, Early Aurignacian and Solutrean/Badegoulian^19^. The Le Piage fauna is largely dominated by reindeer (*Rangifer tarandus*) with rare occurrences of large bovines (*Bos*/*Bison*) and horse (*Equus ferus*). In Montenegro, Crvena Stijena is a large rockshelter with a long sequence of Mousterian and later occupations^20^. The late Mousterian samples from this site show the prevalence of red deer (*Cervus elaphus*), caprines (*Capra ibex/caucasica*) and fallow deer (*Dama dama*)^21^. These sites—with faunal samples dominated by shaft fragments—were selected because they show high levels of fragmentation and relatively low rates of identification (< 10% of the faunal samples), which poses the problem of the representativity of the identified specimens.

Faunal remains from all three sites were identified using the morphological method. In a majority of cases, taxonomic identifications were verified using the reference collection from PACEA (UMR 5199, Université de Bordeaux). The process of identification used a double-blind procedure; the morphological and ZooMS analysts worked independently and unaware of each other’s identifications. No information about taxonomic composition was exchanged between the morphological and ZooMS analysts save for species lists from a few Balkans sites (to the exclusion of Crvena Stijena). To avoid conscious or unconscious biases, piece-plotted specimens were assigned a number and subsampled by a person uninvolved in the project according to a computer-generated list of random numbers. The selected fragments, many of which were morphologically identified specimens, were then anonymized and sent to the ZooMS analyst for collagen fingerprinting. Note that due to budgetary constraints, only a fraction of the morphologically identified specimens—representing between approximately 4–13% of the total NISP at the time of sampling—were examined with ZooMS. Indeterminate specimens that were not piece-plotted were randomly hand-picked from available faunal bags. Once identifications were considered final, results for the two methods were compared; the small number of ZooMS identifications that did not agree with the morphological identifications were re-examined by the ZooMS specialists to evaluate human error (morphological identifications could not, with few exceptions, be reverified due to a lack of access to the material at the time of writing). The results presented here include these minor modifications.

To minimize loss of material, small fragments were removed from the identified remains using a motorized saw. The small size of the removed fragments—most were 10–15 mm on average—created challenges in terms of collagen extraction in sites with poorer faunal preservation. Modifications to a high-throughput ZooMS approach^22^ were carried out which involved a liquid-handling robot (Hamilton, UK)^23^ used to add 0.6 M hydrochloric acid (HCl) to bone samples in 48-well Corning™ Costar™ cell culture-treated flat-bottom plates. These plates were left overnight to decalcify. Following centrifugation at 3700 rpm, 300 µL was removed and added to 96-well ultrafilters and centrifuged at 3700 rpm for 30 min. Then, 500 µL of 50 mM ammonium bicarbonate (AmBiC) was added and once again centrifuged. This centrifugal step was repeated once more, and 100 µL of the retentate removed to a different microtiter plate for digestion. Once this was completed, 0.1 µg sequencing grade trypsin was added, and the plates incubated for 18 h at 37 °C. Lastly, samples were spotted onto a stainless steel MALDI target plate. Spectra were interpreted for known biomarkers published previously (e.g.,^24^), within the *m/z* range 1100–3200. All our identifications can be consulted in the Supplemental spreadsheet 1. Table 1 describes the stratigraphic provenience of the specimens that were selected. In total, 940 remains were examined for collagen fingerprinting, the total per site ranging between 269 (Saint-Césaire) and 360 (Le Piage).

**Table 1:** Provenience and type of specimens used for collagen fingerprinting (ZooMS method).

Layer^1^	Crvena Stijena		
	Identified^2^	Indeterminate^3^	Total
Middle Pal.			
M1	39	41	80
M1/M2	4	3	7
M1/M2a	4	2	6
M2	11		11
M2a	4	1	5
M2b	4	1	5
M2b/M2c2	1		1
M2c/M2c1	12	6	18
M3	6	47	53
M3?	1	1	2
M3/M4	1		1
M3a	1		1
M3b	1	2	3
M4a	5	2	7
M4b	1		1
M4c?		1	1
M5	18	67	85
M5b	1		1
X	6	2	8
XI	2	1	3
XX		2	2
XXIV	7	2	9
XXV	1		1
Total	130	181	311

Layer	Saint-Césaire		
	Identified	Indeterminate	Total
Aurignacian			
US 15		16	16
US 15/16		9	9
US 16	24	136	160
US 16/17		12	12
US 17	42		42
US 17/humus		6	6
M. Pal./Chât.			
US 18	24		24
Total	90	179	269

Layer	Le Piage		
	Identified	Indeterminate	Total
Sol.-Bad.	2	33	35
Late Auri.		4	4
Early Auri.	180	140	320
Auri./Sol.-Bad		1	1
Total	182	178	360

^1^Pal., Paleolithic; M. Pal./Chât., Middle Paleolithic/Châtelperronian; Sol.-Bad., Solutrean/Badegoulian; Auri., Aurignacian.

^2^“Identified” refers to specimens identified to element and taxon using the morphological method.

^3^“Indeterminate” includes small unidentified pieces and specimens that could only be identified to body size class with the morphological method.

In principle, comparisons should focus on identifications made at the same taxonomic level, preferably at least to subtribe or genus. Higher-level taxonomic identifications (e.g., order or family) are less informative due to their low resolution. However, the level of taxonomic precision that can be achieved may vary between the morphological and collagen fingerprinting approaches as a result of method-specific limitations. In the present study, some adjustments were needed given that the identification of some specimens differed slightly in terms of taxonomic rank. Taking estimated body size into account, the “cervine” specimens identified by ZooMS may derive from red deer (*Cervus elaphus*), fallow deer (*Dama dama*) or elk (*Alces alces*). Although all three species are potentially present at Crvena Stijena, the cervine specimens match better in terms of body size with red deer, and thus were attributed to this species. However, the presence of a few remains from the smaller fallow deer among the cervine specimens cannot be excluded (elk provided an unlikely match for the specimens because it is larger than red deer). At Saint-Césaire, the cervine specimen identified by the ZooMS analysts was assigned to red deer because the corresponding morphological identification is consistent with a taxon smaller than elk (fallow deer was absent in the region during the time period documented at the site). The “caprine/*Rangifer*” (ZooMS identifications) from the same site are assumed to be from *Rangifer* as caprines are absent at Saint-Césaire, a pattern consistent with the lack of positively identified *Capra* remains in the associated ZooMS sample and with the rarity of this taxon in similarly dated sites in the region (e.g.,^25^). Note that the results presented here for Saint-Césaire were calculated including and excluding caprine/*Rangifer* identifications. At Crvena Stijena, the “caprine/*Rangifer*” can safely be attributed to *Capra* because the site most probably fell outside the geographical range of reindeer. The fact that this last species has not been positively identified in the study region (e.g.,^26^) supports this interpretation.

Differences in taxonomic distribution between the two methods were evaluated using the chi-square test of independence. We followed Zar’s^27^ recommendations with respect to small cell frequencies in the chi-square tests. To assess variation in taxonomic diversity, the reciprocal of Simpson’s Index was used^28^.

## Results

A relatively high proportion of the morphologically identified specimens at Crvena Stijena (84.6%) and Le Piage (71.4%) could be assigned to a taxon through ZooMS analysis (Table 2), which is indicative of excellent collagen preservation. However, this proportion is considerably lower at Saint-Césaire (51.1%, Table 2). Despite the small dimensions of the finds, a large fraction of the indeterminate, non-piece plotted specimens from Crvena Stijena (44.7%) and Saint-Césaire (81.0%) could likewise be attributed to a taxon using ZooMS.

**Table 2:** Proportions of indeterminate and morphologically identified specimens that could be assigned to a taxon using the ZooMS method.

	Specimens morphologically identified^1^		Indeterminate specimens^2^ (piece-plotted)		Indeterminate specimens (non piece-plotted)		Total sample	
Crvena Stijena	110/130	84.6%	43/49	87.8%	59/132	44.7%	212/311	68.2%
Saint-Césaire	46/90	51.1%			145/179	81.0%	191/269	71.0%
Le Piage	130/182	71.4%	135/178	75.8%			265/360	73.6%
Total	286/402	71.1%	178/227	78.4%	204/311	65.6%	668/940	71.1%

^1^ZooMS identifications include cases where the taxon is imprecise (i.e., one specimen with the mention of “Reptilia?” at Crvena Stijena). “Caprine/*Rangifer*” specimens were treated as “caprines” at Crvena Stijena and as “*Rangifer*” at Saint-Césaire based on the range distribution of these taxa (see text for details).

^2^The “piece-plotted” specimens sampled for this study typically measured 50–100 mm whereas the non-piece plotted specimens were generally < 25 mm.

### Patterns in the NISP sample

**Figure 1:**
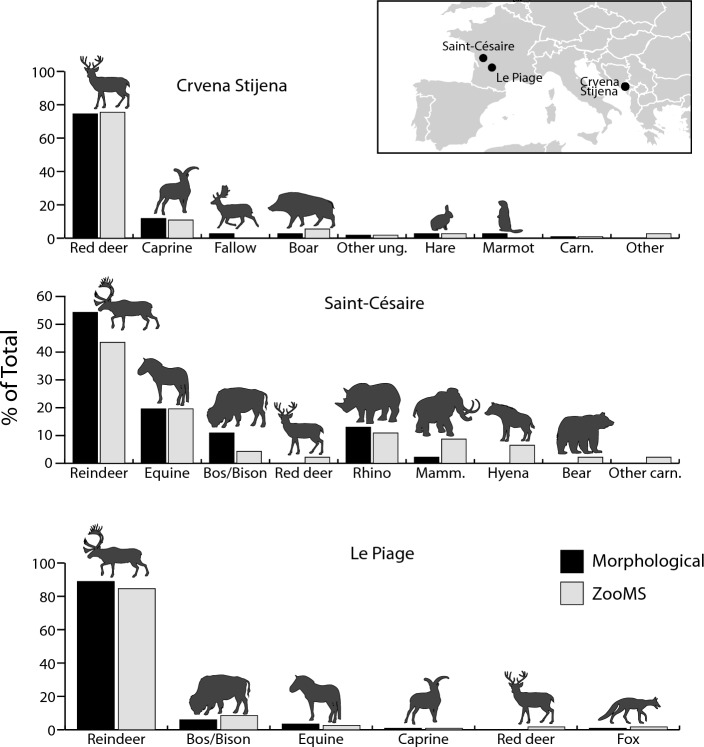
Comparison of taxonomic composition between the morphological and ZooMS identifications. The inset shows the location of the sites. Data from Table S1. Other ung., other ungulates; Mamm., mammoth; Carn., carnivores. The inset was created in Adobe Illustrator 2020.

Because they provide the foundation of a large number of faunal interpretations, our comparisons begin with an analysis of the NISP sample (all layers are combined here to increase sample size, an analysis by layer is presented below). A chi-square test of independence shows no statistical difference in the taxonomic profiles produced by the two methods at all three sites (Crvena Stijena: χ^2^ = 14.0, *p* = 0.1709; Saint-Césaire: χ^2^ = 9.7, *p* = 0.2843; Le Piage: χ^2^ = 3.1, *p* = 0.6801, Fig. 1, data from Table S1), an indication that the counts are not affected by the method of identification. Out of 26 pairwise comparisons of taxa, only one shows a taxonomic difference larger than 7% (reindeer at Saint-Césaire, difference of 10.8% between the two methods, Table S1).

**Figure 2:**
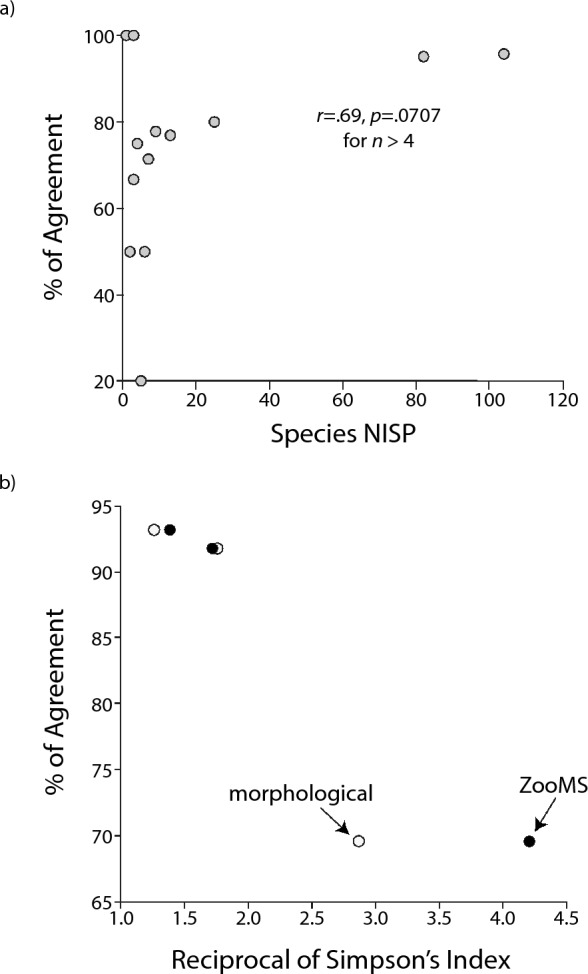
Relationship between the percentage of agreement and (**a**) the NISP sample size for a species, and (**b**) the Reciprocal of Simpson’s Index. Values are calculated by species in (**a**) and for the whole assemblage in (**b**). Data from Table 3.

Because errors may cancel one another in comparisons of whole samples, an additional test consists in determining if the morphological and ZooMS identifications are consistent for the same specimen. In this case, we are ascertaining whether a given morphological identification—a *Bos*/*Bison* femur for instance—matches the taxon given by ZooMS for the same specimen. The percentage of agreement, that is, the proportion of matches relative to the sum of compared specimens, is moderate at Saint-Césaire (69.6%), whereas it is high at Crvena Stijena (91.8%) and Le Piage (93.2%, Table 3). A plot of the data suggests that errors of identification are more common when species are poorly represented, although the trend is not significant (*r* = 0.69, *p* = 0.0707, Fig. 2a, *n* > 4). The percentage of agreement in our dataset also seems inversely correlated with species diversity as measured by the reciprocal of Simpson’s Index, a pattern that is observed regardless of whether the identifications are based on the morphological or ZooMS counts (Fig. 2b).

**Table 3:** Agreement in taxonomic attribution between the morphological and collagen fingerprinting methods.

Morphological	Crvena Stijena		Saint-Césaire^2^		Le Piage		Total	
	Agree?^1^	%Agree	Agree?	%Agree	Agree?	%Agree	Agree?	%Agree
*Bos*/*Bison*			1/5	20.0	5/7	71.4	6/12	50.0
*Capra* sp.	10/13	76.9			1/1	100	11/14	78.6
*Capreolus* sp.	1/2	50.0					1/2	50.0
*Equus* sp.			7/9	77.8	3/4	75.0	10/13	76.9
*Cervus elaphus*	78/82	95.1					78/82	95.1
*Dama dama*	2/3	66.7					2/3	66.7
Leporidae	3/3	100					3/3	100
*Mammuthus* sp.			1/1	100			1/1	100
*Marmota* sp.	3/3	100					3/3	100
*Rangifer tarandus*			20/25 (13/18)	80.0 (72.2)	99/104	95.7	119/129	92.2
*Panthera* sp.	1/1	100					1/1	100
Rhinocerotidae			3/6	50.0			3/6	50.0
*Sus scrofa*	3/3	100					3/3	100
*Vulpes* sp.					1/1	100	1/1	100
Total	101/110	91.8	32/46 (25/39)	69.6 (64.1)	109/117	93.2	242/273	88.6

^1^The “Agree?” column shows the proportion of morphologically identified specimens that gave a similar identification when examined with the collagen fingerprinting method. The frequencies are converted into percentages in the “%Agree” column. Specimens with imprecise ZooMS identifications—most are specimens that could only be attributed to a body size class—are excluded (see Table 2 and text for methodological details). These less precise identifications are generally consistent with the associated ZooMS identifications.

^2^The numbers in parentheses exclude specimens identified by ZooMS as caprine/*Rangifer.*

**Figure 3:**
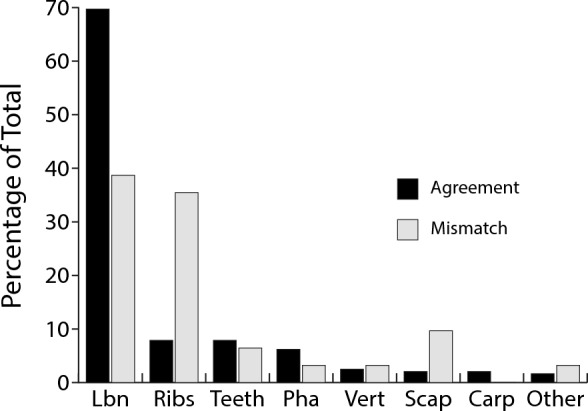
Comparison of patterns of skeletal representation between the morphological and ZooMS identifications. Data from Table S2, residuals are given in Table S3. Lbn, long bones; Pha, phalanges; Vert, vertebrae; Scap, scapula; Carp, carpals/tarsals.

Turning to patterns of skeletal representation, a chi-square test of independence and the adjusted standardized residuals show a clear over-representation of ribs, and to a lesser extent, humeri and scapulae in the mismatch sample (χ^2^ = 31.2, *p* < 0.001, Fig. 3, categories for the test as in the histogram), the results showing a moderate effect size (Cramér’s V = 0.35). If ribs are excluded from the NISP sample, the percentage of agreement is substantially increased at Saint-Césaire (from 69.6 to 82.3%) where this body part is unusually abundant (63.0% in the sample examined here vs. 0% at Crvena Stijena and 0.9% at Le Piage). Conversely, long bones are less commonly represented in the mismatch sample.

### Comparing the NISP sample with the indeterminate remains

**Figure 4:**
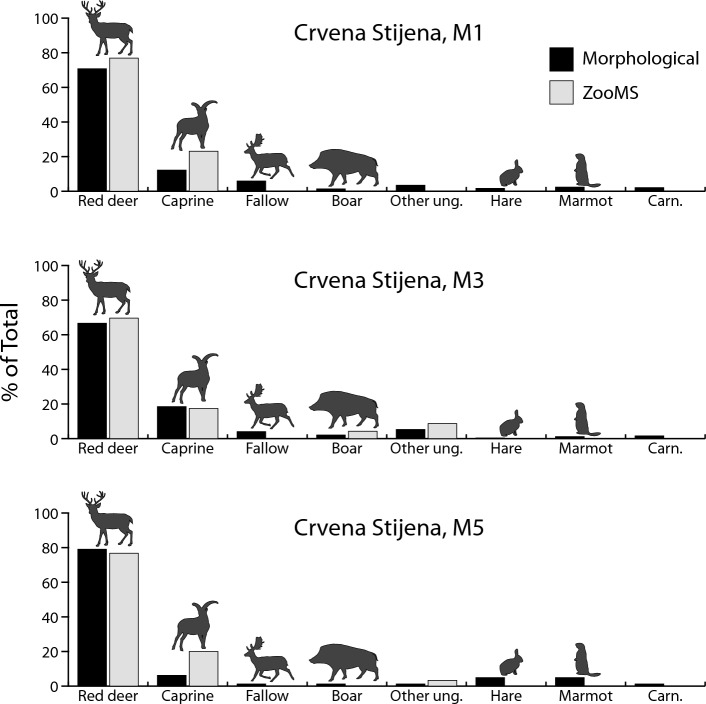
Comparison of morphological identifications in three layers at Crvena Stijena with ZooMS identifications for fragments that could not be identified using the morphological approach. Data from Table S4. M5 is the oldest, and M1 the youngest, layer, respectively. Abbreviations as in Fig. 1.

**Figure 5:**
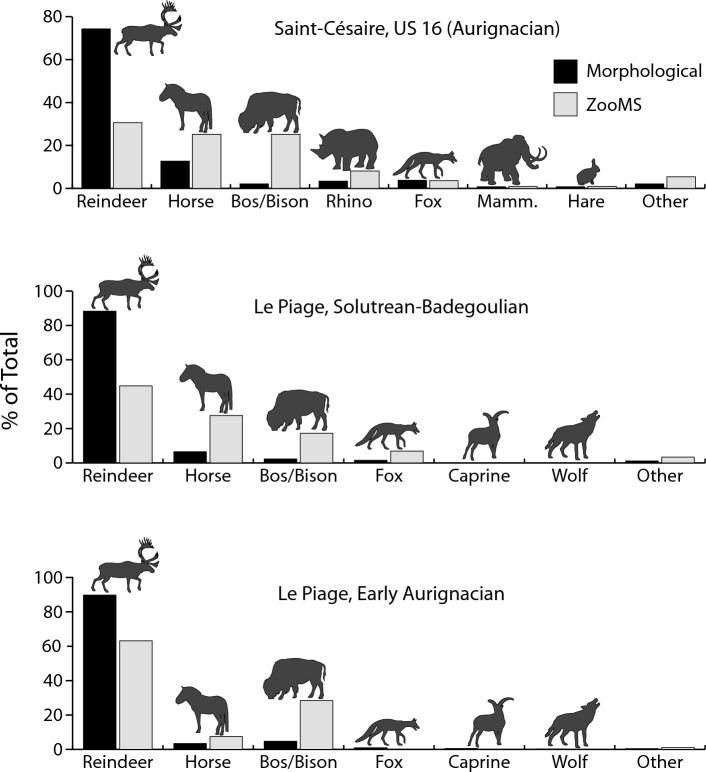
Comparison of morphological identifications at Saint-Césaire and Le Piage with ZooMS identifications for fragments that could not be identified using the morphological approach. Data from Table S5.

While the two methods give comparable results for the NISP sample, what remains to be determined is whether results are also consistent when comparisons are performed with the indeterminate sample (specimens in this sample lack a morphological identification). At Crvena Stijena, comparing ZooMS identifications for morphologically indeterminate specimens with published NISP counts for the M5, M3 and M1 levels—three Middle Paleolithic occupations dated to Marine Isotope Stage 3—yields only small differences that are not statistically significant (M5: χ^2^ = 8.8, *p* = 0.2637; M3: χ^2^ = 4.9, *p* = 0.9612; M1: χ^2^ = 6.7, *p* = 0.8751, data from Table S4, Fig. 4). Unlike Crvena Stijena, the taxonomic abundances in the NISP sample at Saint-Césaire and Le Piage are significantly different from the ZooMS counts derived from the indeterminate sample (Saint-Césaire, US 16: χ^2^ = 92.2, *p* < 0.0001; Le Piage, Early Aurignacian: χ^2^ = 110.1, *p* < 0.0001; Le Piage, Solutrean/Badegoulian: χ^2^ = 71.0, *p* < 0.0001, data from Table S5). The results for these comparisons show a moderate to strong effect size (Saint-Césaire, US 16: Cramér’s V = 0.51; Le Piage, Early Aurignacian: Cramér’s V = 0.20; Le Piage, Solutrean/Badegoulian: Cramér’s V = 0.16). This lack of agreement means that, despite a similar array of species, the indeterminate sample contains proportions of taxa that are different from those of the NISP samples, with large species such as horse, *Bos*/*Bison* and rhinoceros being systematically more common in the indeterminate fraction (Fig. 5). These differences in taxonomic proportions are confirmed by a chi-square comparison of the ZooMS counts for the NISP and indeterminate samples in the Early Aurignacian at Le Piage (χ^2^ = 19.9, *p* = 0.0013, Cramér’s V = 0.31, data from Table S6, note that the ZooMS counts by layer at the other sites are too small to allow direct comparisons between the NISP and indeterminate samples).

Although the indeterminate samples are smaller for the other units, the ZooMS analysis provides us with valuable qualitative information on species that are rare or otherwise undocumented in the selected samples. For instance, one hominin remain was identified by ZooMS in unit US 15/16 at Saint-Césaire, an important finding given the scarcity of human remains for the early Upper Paleolithic (see Supplemental spreadsheet S1). Other rare species identified by ZooMS in the course of this study include one specimen attributed to *Panthera* (US 15, Saint-Césaire). The identification of these new species is unsurprising given that larger samples are frequently associated with increased taxonomic diversity (e.g.,^29,30^).

## Discussion and conclusion

The comparisons between the two methods are encouraging for the NISP sample because they are consistent at all three sites. However, there are some hints that rare species may be more commonly misidentified by the morphological method than those that are common. A possible explanation is that analysts using the morphological approach may occasionally overlook rare taxa while identifying specimens, an issue that has been raised in previous research^2,31^. Whether this problem also affects the ZooMS identifications is possible but cannot be confirmed. Despite some hurdles with respect to collagen extraction, the use of small fragments, with presumably smaller amounts of preserved organic material, does not appear to have overly affected the ZooMS identifications. At Saint-Césaire, the percentage of agreement is substantially lower than at the other sites. Although this may be due to its more diverse fauna, the high percentage of ribs in the Saint-Césaire sample—a body part notoriously difficult to identify to taxon^8^—likely accounts for the lower percentage of agreement observed at this site.

Comparisons with the indeterminate sample showed mixed results. While no significant differences were observed at Crvena Stijena, the indeterminate samples from Saint-Césaire and Le Piage contain a markedly higher proportion of large game than the NISP samples from the same sites. Other studies in Europe have noted an over-representation of large game with the ZooMS method, a pattern that was attributed to differential processing^32,33^. Several factors may account for the over-representation of large game in the ZooMS samples at these sites. At Saint-Césaire, bone fragments from larger fauna were argued to be under-identified because the original skeletal elements tends to be more fragmented relative to those from smaller species^34^. Also relevant is the problem of specimen interdependence, which arises when the units that are counted derive from the same element^30^. The fact that the indeterminate specimens in our sample were collected from a small number of provenience bags might have accentuated the problem of specimen interdependence as the bones from large taxa are more susceptible to in situ post-depositional fragmentation because they contain a higher proportion of cancellous bone, a fragile tissue, than those of smaller taxa. For these reasons, determining whether the under-representation of large species in the NISP sample at Saint-Césaire and Le Piage is real or a methodological artifact is difficult to ascertain. Our impression is that specimens from large taxa are both under-represented in the NISP sample and over-represented in the indeterminate sample as a consequence of an increased degree of fragmentation and a concomitant lower probability of identification in comparison to smaller fauna.

The fact that the ZooMS method can be used to identify indeterminate fragments to taxon represents a significant advance because it allows us to better appreciate the impact of fragmentation on patterns of taxonomic representation. Whereas subjectivity is often perceived as being specific to the morphological approach, our results also highlight the need for caution in manually identifying spectra using collagen fingerprinting (e.g., *Rangifer* vs. *Capra* where the ‘A’ marker is poor, or masked by nearby peaks), which can yield errors; in this case we observed more errors than expected due to a relatively greater amount of ‘inorganic’ peaks appearing close to (within 1 Da) the *m/z* value of some markers, a problem accentuated by the small size of the fragments used in this study (specimens analyzed by ZooMS are typically larger in other studies). Potential for human-derived error currently remains in the ZooMS process, as with most scientific applications, including (mis)labeling problems and errors in marker selection. The latter is likely to be reduced with the increasing use of machine learning and other computer-derived forms of fingerprint identification (e.g.,^35,36^). Nonetheless, machine learning also has its limits; in contexts of imperfect collagen preservation—as is the case at Saint-Césaire and Le Piage—low-quality data may prevent a full use of its potential for distinguishing problematic peaks. Given that they can be used for cross-validation, our data emphasize the importance of identifying remains using both morphological and collagen fingerprinting methods. Despite the advances afforded by ZooMS, the morphological method remains critical as it can yield valuable taxonomic information when collagen is poorly preserved or when a specimen belongs to a species whose markers are not well resolved.

As it provides an independent and cost-effective alternative to morphological identification, ZooMS improves our understanding of the geographical distribution of species in the past and expands our knowledge of the impact of carcass processing on taxonomic identification. Because errors can be corrected and new species identified, archaeozoologists have much to learn by comparing their results with independently generated identifications. For this reason, we strongly encourage the use of collagen fingerprinting methods in faunal analysis.

### Supplementary Information

Supplementary Tables.

Supplementary Information 2.

## Data Availability

All data are provided in the Supplemental spreadsheet 1.
