# Supplementary material for: A double-blind comparison of morphological and collagen fingerprinting (ZooMS) methods of skeletal identifications from Paleolithic contexts
Source: Sci Rep. 2023 Nov 1;13:18825. doi: 10.1038/s41598-023-45843-4 (PMC10620384; doi:10.1038/s41598-023-45843-4)
Supplement: Supplementary file 1 — Supplementary Tables. [file 41598_2023_45843_MOESM1_ESM.docx]

|  | Crvena Stijena | | | | Saint-Césaire | | | | Le Piage | | | |
| --- | --- | --- | --- | --- | --- | --- | --- | --- | --- | --- | --- | --- |
|  | Morph. | | ZooMS | | Morph. | | ZooMS | | Morph. | | ZooMS | |
|  | *n* | % | *n* | % | *n* | % | *n* | % | *n* | % | *n* | % |
| Ungulates |  |  |  |  |  |  |  |  |  |  |  |  |
| *Rangifer tarandus* |  |  |  |  | 25 | 54.3 | 20 | 43.5 | 104 | 88.9 | 99 | 84.6 |
| Equine |  |  |  |  | 9 | 19.6 | 9 | 19.6 | 4 | 3.4 | 3 | 2.6 |
| *Bos*/*Bison* |  |  | 1 | 0.9 | 5 | 10.9 | 2 | 4.3 | 7 | 6.0 | 10 | 8.5 |
| *Cervus elaphus* | 82 | 74.5 | 83 | 75.5 |  |  | 1 | 2.2 |  |  | 2 | 1.7 |
| *Capra* sp. | 13 | 11.8 | 12 | 10.9 |  |  |  |  | 1 | 0.9 | 1 | 0.9 |
| Rhinocerotidae |  |  |  |  | 6 | 13.0 | 5 | 10.9 |  |  |  |  |
| *Mammuthus* |  |  |  |  | 1 | 2.2 | 4 | 8.7 |  |  |  |  |
| *Dama dama* | 3 | 2.7 |  |  |  |  |  |  |  |  |  |  |
| *Sus scrofa* | 3 | 2.7 | 6 | 5.5 |  |  |  |  |  |  |  |  |
| *Capreolus capr.* | 2 | 1.8 |  |  |  |  |  |  |  |  |  |  |
| *Ovis* sp. |  |  | 1 | 0.9 |  |  |  |  |  |  |  |  |
| Carnivores |  |  |  |  |  |  |  |  |  |  |  |  |
| *Panthera* sp. | 1 | 0.9 | 1 | 0.9 |  |  | 1 | 2.2 |  |  |  |  |
| *Vulpes* sp. |  |  |  |  |  |  |  |  | 1 | 0.9 | 2 | 1.7 |
| Hyaenidae |  |  |  |  |  |  | 3 | 6.5 |  |  |  |  |
| *Ursus* sp. |  |  |  |  |  |  | 1 | 2.2 |  |  |  |  |
| Other taxa |  |  |  |  |  |  |  |  |  |  |  |  |
| Lagomorph | 3 | 2.7 | 3 | 2.7 |  |  |  |  |  |  |  |  |
| *Marmota* sp. | 3 | 2.7 |  |  |  |  |  |  |  |  |  |  |
| Rodentia |  |  | 3 | 2.7 |  |  |  |  |  |  |  |  |
| Total | 110 | 100.0 | 110 | 100.0 | 46 | 100.0 | 46 | 100.0 | 117 | 100.0 | 117 | 100.0 |

Table S1. Taxonomic counts for the specimens that were identified using both the morphological and collagen fingerprinting methods. Only specimens that yielded results for both methods are included in this table.

|  | Agreement^1^ | | Mismatch | |  | Agreement | | Mismatch | |
| --- | --- | --- | --- | --- | --- | --- | --- | --- | --- |
| Part | *n* | % | *n* | % | Part | *n* | % | *n* | % |
| Femur | 32 | 13.3 |  |  | Humerus | 14 | 5.8 | 5 | 16.1 |
| Metatarsal | 29 | 12.0 | 3 | 9.7 | Vertebrae | 6 | 2.5 | 1 | 3.2 |
| Ribs | 19 | 7.9 | 11 | 35.5 | Scapula | 5 | 2.1 | 3 | 9.7 |
| Tibia | 26 | 10.8 | 1 | 3.2 | Carpals/tarsals | 5 | 2.1 |  |  |
| Radio-ulna | 25 | 10.4 | 1 | 3.2 | Innominate | 2 | 0.8 |  |  |
| Metacarpal | 22 | 9.1 | 1 | 3.2 | Fibula | 1 | 0.4 |  |  |
| Metapodial | 20 | 8.3 | 1 | 3.2 | Sacrum | 1 | 0.4 |  |  |
| Mandible/maxillary | 19 | 7.9 | 2 | 6.5 | Antler |  |  | 1 | 3.2 |
| Phalanges | 15 | 6.2 | 1 | 3.2 | Total^2^ | 241 | 100 | 31 | 100 |

^1^“Agreement” means that the morphological and ZooMS approaches are in agreement with respect to taxonomic identification, whereas “Mismatch” means that the taxon is inconsistent between the two methods. All identifications are combined to increase sample size.

^2^The skeletal information was erroneous for one specimen, which explains why the total is one specimen less than in Table 3.

Table S2. Skeletal representation in the agreement and mismatch samples.

|  | Agreement | Mismatch |  | Agreement | Mismatch |
| --- | --- | --- | --- | --- | --- |
| Femur | **2.16** | **-2.16** | Mandible/maxillary | 0.28 | -0.28 |
| Metatarsal | 0.38 | -0.38 | Phalanges | 0.67 | -0.67 |
| Ribs | **-4.62** | **4.62** | Humerus | **-2.12** | **2.12** |
| Tibia | 1.33 | -1.33 | Vertebrae | -0.24 | 0.24 |
| Radio-ulna | 1.27 | -1.27 | Scapula | **-2.36** | **2.36** |
| Metacarpal | 1.11 | -1.11 | Carpals/tarsals | 0.81 | -0.81 |
| Metapodial | 1.00 | -1.00 | Other | -0.61 | 0.61 |

Table S3. Table of adjusted standardized residuals comparing the representation of skeletal parts in the agreement and mismatch samples. Large residuals (shown in bold) are significantly different from their expected frequency. Data from Table S2.

|  | M5 | | | |  | M3 | | | |  | M1 | | | |
| --- | --- | --- | --- | --- | --- | --- | --- | --- | --- | --- | --- | --- | --- | --- |
|  | Morphological | | ZooMS | |  | Morphological | | ZooMS | |  | Morphological | | ZooMS | |
| Ungulates | *n* | % | *n* | % |  | *n* | % | *n* | % |  | *n* | % | *n* | % |
| *Cervus elaphus* | 64 | 79.0 | 23 | 76.7 |  | 162 | 66.7 | 16 | 69.6 |  | 204 | 70.8 | 20 | 76.9 |
| *Capra* sp. | 5 | 6.2 | 6 | 20.0 |  | 45 | 18.5 | 4 | 17.4 |  | 35 | 12.2 | 6 | 23.1 |
| *Dama dama* | 1 | 1.2 |  |  |  | 10 | 4.1 |  |  |  | 17 | 5.9 |  |  |
| *Bos*/*Bison* | 1 | 1.2 | 1 | 3.3 |  | 7 | 2.9 | 2 | 8.7 |  | 6 | 2.1 |  |  |
| *Equus ferus* |  |  |  |  |  | 4 | 1.6 |  |  |  | 2 | 0.7 |  |  |
| *Sus scrofa* | 1 | 1.2 |  |  |  | 5 | 2.1 | 1 | 4.3 |  | 4 | 1.4 |  |  |
| *Rupicapra rup*. |  |  |  |  |  | 2 | 0.8 |  |  |  | 1 | 0.3 |  |  |
| *Capreolus capr*. |  |  |  |  |  |  |  |  |  |  | 1 | 0.3 |  |  |
| Carnivores |  |  |  |  |  |  |  |  |  |  |  |  |  |  |
| *Canis*/*Cuon* | 1 | 1.2 |  |  |  |  |  |  |  |  | 4 | 1.4 |  |  |
| *Ursus* sp. |  |  |  |  |  | 1 | 0.4 |  |  |  | 1 | 0.3 |  |  |
| *Vulpes* sp. |  |  |  |  |  | 1 | 0.4 |  |  |  | 1 | 0.3 |  |  |
| *Panthera pardus* |  |  |  |  |  | 1 | 0.4 |  |  |  |  |  |  |  |
| *Lynx* sp. |  |  |  |  |  | 1 | 0.4 |  |  |  |  |  |  |  |
| Other taxa |  |  |  |  |  |  |  |  |  |  |  |  |  |  |
| leporids | 4 | 4.9 |  |  |  | 1 | 0.4 |  |  |  | 5 | 1.7 |  |  |
| *Marmota marm*. | 4 | 4.9 |  |  |  | 3 | 1.2 |  |  |  | 7 | 2.4 |  |  |
| Total | 81 | 100.0 | 30 | 100.0 |  | 243 | 100.0 | 23 | 100.0 |  | 288 | 100.0 | 26 | 100.0 |

Table S4. Comparison of published morphological identifications at Crvena Stijena (Morin and Soulier 2017) with ZooMS identifications for fragments that could not be identified using the morphological approach. M5 is the oldest, and M1 the youngest, layer, respectively.

|  | Saint-Césaire, US 16 | | | |  | Le Piage, Early Auri. | | | |  | Le Piage, Sol.-Bad. | | | |
| --- | --- | --- | --- | --- | --- | --- | --- | --- | --- | --- | --- | --- | --- | --- |
|  | Morphological | | ZooMS | |  | Morphological | | ZooMS | |  | Morphological | | ZooMS | |
| Ungulates | *n* | % | *n* | % |  | *n* | % | *n* | % |  | *n* | % | *n* | % |
| *Rangifer tarandus* | 176 | 74.3 | 34 | 30.6 |  | 2290 | 89.8 | 60 | 63.2 |  | 2341 | 88.3 | 13 | 44.8 |
| Equine | 30 | 12.7 | 28 | 25.2 |  | 87 | 3.4 | 7 | 7.4 |  | 172 | 6.5 | 8 | 27.6 |
| *Bos*/*Bison* | 5 | 2.1 | 28 | 25.2 |  | 121 | 4.7 | 27 | 28.4 |  | 60 | 2.3 | 5 | 17.2 |
| *Cervus elaphus* |  |  | 2 | 1.8 |  | 3 | 0.1 | 1 | 1.1 |  | 5 | 0.2 | 1 | 3.4 |
| *Megaloceros gig.* | 2 | 0.8 |  |  |  |  |  |  |  |  |  |  |  |  |
| *Capra* sp. |  |  |  |  |  | 12 | 0.5 | 0 | 0 |  | 3 | 0.1 |  |  |
| Rhinocerotidae | 8 | 3.4 | 9 | 8.1 |  |  |  |  |  |  |  |  |  |  |
| *Mammuthus* | 2 | 0.8 | 1 | 0.9 |  | 1 | 0 |  |  |  |  |  |  |  |
| *Sus scrofa* |  |  | 0 | 0 |  |  |  |  |  |  | 7 | 0.3 |  |  |
| *Capreolus capr.* |  |  |  |  |  | 1 | 0 |  |  |  | 1 | 0 |  |  |
| *Rupicapra rupi.* |  |  |  |  |  |  |  |  |  |  | 1 | 0 |  |  |
| *Saiga tatarica* |  |  |  |  |  |  |  |  |  |  | 2 | 0.1 |  |  |
| Carnivores |  |  |  |  |  |  |  |  |  |  |  |  |  |  |
| *Panthera* sp. |  |  |  |  |  |  |  |  |  |  |  |  |  |  |
| *Vulpes* sp. | 9 | 3.8 | 4 | 3.6 |  | 23 | 0.9 |  |  |  | 40 | 1.5 | 2 | 6.9 |
| *Canis lupus* | 2 | 0.8 |  |  |  | 5 | 0.2 |  |  |  | 6 | 0.2 |  |  |
| *Ursus* sp. |  |  | 1 | 0.9 |  |  |  |  |  |  |  |  |  |  |
| *Mustela putorius* | 1 | 0.4 |  |  |  |  |  |  |  |  |  |  |  |  |
| *Lynx/Felis* |  |  |  |  |  |  |  |  |  |  | 6 | 0.2 |  |  |
| *Crocuta crocuta* |  |  |  |  |  |  |  |  |  |  | 1 | 0 |  |  |
| Other taxa |  |  |  |  |  |  |  |  |  |  |  |  |  |  |
| Lagomorph | 2 | 0.8 | 1 | 0.9 |  |  |  |  |  |  |  |  |  |  |
| Bird |  |  | 3 | 2.7 |  | 6 | 0.2 |  |  |  | 7 | 0.3 |  |  |
| Total | 237 | 100.0 | 111 | 100.0 |  | 2549 | 100.0 | 95 | 100.0 |  | 2652 | 100.0 | 29 | 100.0 |

Table S5. Comparison of morphological identifications at Saint-Césaire and Le Piage with ZooMS identifications for fragments that could not be identified using the morphological approach. The Early Aurignacian is an early Upper Paleolithic industry whereas the Solutrean-Badegoulian is a mixed sample dating to the later Upper Paleolithic. “US 16” at Saint-Césaire is associated with an Early/Middle Aurignacian industry.

|  | Le Piage, Early Auri. | | | |
| --- | --- | --- | --- | --- |
|  | NISP | | Indeterminate | |
| Ungulates | *n* | % | *n* | % |
| *Rangifer tarandus* | 98 | 84.5 | 60 | 63.2 |
| Equine | 3 | 2.6 | 7 | 7.4 |
| *Bos*/*Bison* | 10 | 8.6 | 27 | 28.4 |
| *Cervus elaphus* | 2 | 1.7 | 1 | 1.1 |
| *Capra* sp. | 1 | 0.9 |  |  |
| *Vulpes* sp. | 2 | 1.7 |  |  |
| Total | 116 | 100.0 | 95 | 100.0 |

Table S6. Comparison of ZooMS taxonomic counts for the NISP and indeterminate samples in the Early Aurignacian sample at Le Piage.
